# Mutagenicity and antimutagenicity of (−)-hinokinin a trypanosomicidal compound measured by *Salmonella* microsome and comet assays

**DOI:** 10.1186/1472-6882-12-203

**Published:** 2012-10-31

**Authors:** Flávia Aparecida Resende, Lilian Cristina Barbosa, Denise Crispim Tavares, Mariana Santoro de Camargo, Karen Cristina de Souza Rezende, Márcio Luis de Andrade e Silva, Eliana Aparecida Varanda

**Affiliations:** 1 Departamento de Ciências Biológicas, UNESP-Universidade Estadual Paulista Julio de Mesquita Filho- Faculdade de Ciências Farmacêuticas de Araraquara, Araraquara, São Paulo, 14801-902, Brazil; 2 Universidade de Franca, Franca, São Paulo, 14404-600, Brazil

**Keywords:** Hinokinin, Ames test, Comet assay, Mutagenicity, Antimutagenicity

## Abstract

**Background:**

The dibenzylbutyrolactone lignan (−)-hinokinin (HK) was derived by partial synthesis from (−)-cubebin, isolated from the dry seeds of the pepper, *Piper cubeba*. Considering the good trypanosomicidal activity of HK and recalling that natural products are promising starting points for the discovery of novel potentially therapeutic agents, the aim of the present study was to investigate the (anti) mutagenic∕ genotoxic activities of HK.

**Methods:**

The mutagenic∕ genotoxic activities were evaluated by the Ames test on *Salmonella typhimurium* strains TA98, TA97a, TA100 and TA102, and the comet assay, so as to assess the safe use of HK in the treatment of Chagas’ disease. The antimutagenic ∕antigenotoxic potential of HK were also tested against the mutagenicity of a variety of direct and indirect acting mutagens, such as 4- nitro-*o*-phenylenediamine (NOPD), sodium azide (SA), mitomycin C (MMC), benzo[*a*]pyrene (B[*a*]P), aflatoxin B_1_ (AFB_1_), 2-aminoanthracene (2-AA) and 2-aminofluorene (2-AF), by the Ames test, and doxorubicin (DXR) by the comet assay.

**Results:**

The mutagenicity∕genotoxicity tests showed that HK did not induce any increase in the number of revertants or extent of DNA damage, demonstrating the absence of mutagenic and genotoxic activities. On the other hand, the results on the antimutagenic potential of HK showed a strong inhibitory effect against some direct and indirect-acting mutagens.

**Conclusions:**

Regarding the use of HK as an antichagasic drug, the absence of mutagenic effects in animal cell and bacterial systems is encouraging. In addition, HK may be a new potential antigenotoxic ∕ antimutagenic agent from natural sources. However, the protective activity of HK is not general and varies with the type of DNA damage-inducing agent used.

## Background

**Figure 1:**
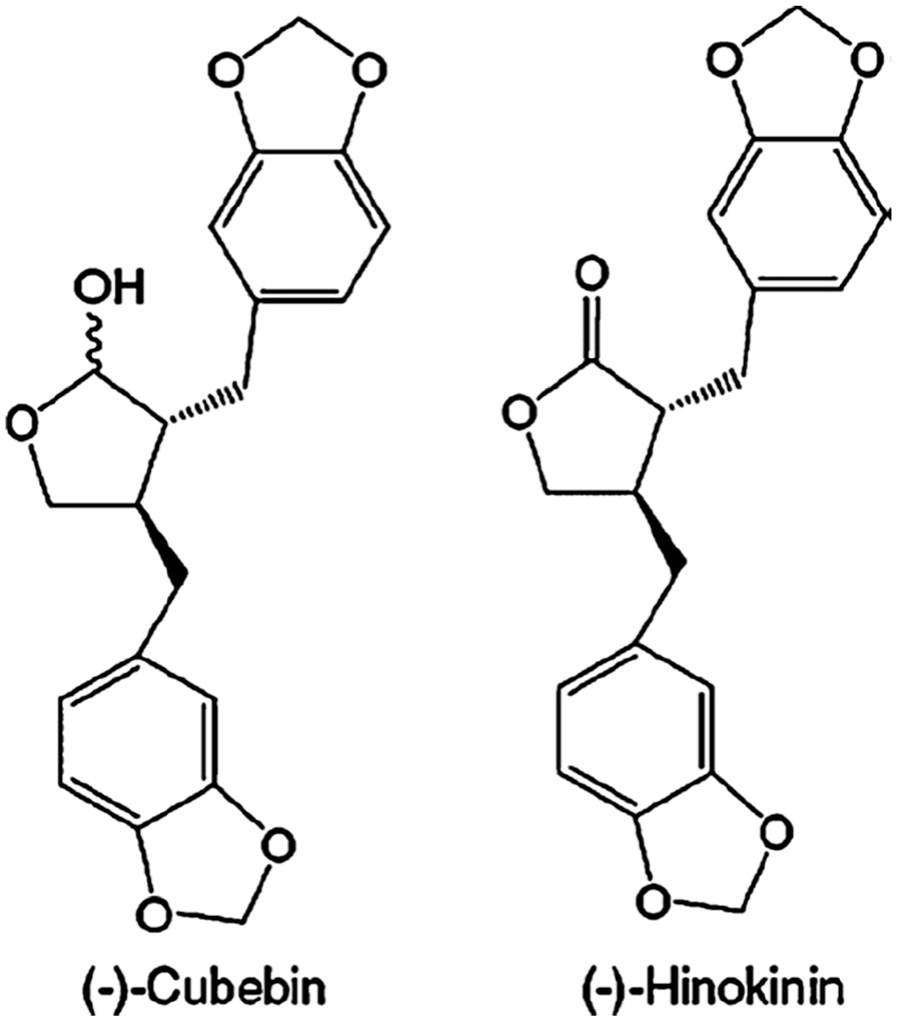
**Chemical structures of (−)-cubebin and (−)-hinokinin**.

(−)-Hinokinin (HK – Figure 1), a dibenzylbutyrolactone lignan, was derived by partial synthesis from (−)-cubebin (Figure 1) isolated from the dry seeds of *Piper cubeba*[1] and proved to be a potential candidate for the development of a new drug to treat Chagas’ disease [2,3]. The drugs currently used to treat Chagas’ disease are two nitroheterocyclic drugs, the nitrofuran nifurtimox (Lampit^®^), whose production has now been discontinued, and the 2-nitroimidazole benznidazole (Rochagan^®^) [4]. These drugs have demonstrated several limitations in use, in part due to their low bioavailability, their limited efficacy against the various stages of the disease and the development of parasite resistance. The other main contraindication of both drugs is their significant toxicity [5]. 

The most frequent side effects of these drugs include anorexia, vomiting, peripheral polyneuropathy and allergic dermopathy. *In vivo* toxic effects and mutagenicity have been clearly proved in the case of nifurtimox [6]. Also, benznidazole has exhibited genotoxic effects *in vitro*[7] and significant *in vivo* alterations [8]. For this reason, the development of safer and more effective drugs for Chagas’ disease is an urgent priority [5].

Studies have shown that HK has higher trypanosomicidal activity than benznidazole against epimastigote forms and a similar activity against amastigote forms [2,9], which aroused considerable scientific interest in this lignan. Moreover, HK exhibits activity against oral pathogens, including *Streptococcus mutans*[10], antioxidant activity *in vitro*[1], analgesic and anti-inflammatory activities [11], as well as antimutagenic activity by the micronucleus test, *in vivo*[1] and *in vitro*[12].

In light of the good trypanosomicidal activity of HK and given that natural products are promising sources of novel potentially therapeutic agents, the aim of the present study was to investigate its mutagenic and genotoxic activities by the Ames and comet assays, respectively, to assess the safety of using HK in the treatment of Chagas’ disease. In the absence of such activity, the antimutagenic and antigenotoxic potential would also be tested, with a view to discovering antiparasite agents that can protect the genetic material against damage.

## Methods

### Isolation of (−)-cubebin

Powdered seeds from commercially available *Piper cubeba* L. fruits were exhaustively extracted by maceration with 96%; ethanol. The crude extract was concentrated by evaporation and partitioned between hexane and methanol/water (9:1) phases, providing 430 g of the dried methanol/water fraction. This mass was submitted to repeated column chromatography on 1.0 kg silica gel (12 × 120 cm). The cubebin-rich fractions (hexane/dichloromethane 1:1 and 100%; dichloromethane) were subjected to repeated crystallization in hexane/acetone to provide crystalline (−)-cubebin (37 g), mp 130–131°C, [α]_*D*_^26^–8.12° (*c* 0.46, CHCl_3_). The chemical structure was confirmed by ^1^H NMR and IR, by comparison with published data [13]. Purity was estimated to be 99%; by both HPLC and spectral data analysis. 

### Preparation of (−)-hinokinin

(−)-Cubebin (0.5004 g in 10 mL dichloromethane) was treated with two equivalents (2.32 mM) of pyridinium chlorochromate at room temperature and the reaction mixture was stirred for 12 h. The solvent was removed under vacuum and the residue was submitted to chromatography on silica gel, eluted with hexane-ethyl acetate (4:1), yielding 0.4926 g (98%;) of an oily product ([α]_*D*_^26^–30 (26°C) (*c* 0.99, CHCl_3_)): ^1^H-NMR δ (CDCl_3_) 6.8-6.4 (m, 1 H), 5.9 (sl, 2 H), 4.15 (dd, 1 H, *J* = 7.1 Hz and *J* = 9.3 Hz), 3.85 (dd, 1 H, *J* = 7.1 Hz and *J* = 9.1 Hz), 3.0 (dd, 1 H, *J* = 5.1 Hz and *J* = 14.2 Hz), 2.85 (dd, 1 H, *J* = 7.3 Hz and *J* = 14.2 Hz), 2.6 (d, 1 H, *J* = 7.1 Hz), 2.55 (m, 1 H), 2.45(d, 1 H, *J* = 8,6 Hz), 2.4 (m, 1 H); ^13^C-NMR δ (CDCl_3_) 178.4, 147.9, 147.8, 146.5, 146.4, 131.6, 131.3, 122.2, 121.55, 109.4, 108.8, 108.4, 108.3, 101.0, 71.2, 46.4, 41.3, 38.4, 34.8 [9].

### Chemicals and culture media

Dimethylsulfoxide (DMSO), nicotinamide adenine dinucleotide phosphate sodium salt (NADP), D-glucose-6-phosphate disodium salt, magnesium chloride, L-histidine monohydrate, D-biotin, 4-nitro-*o*-phenylenediamine (NOPD), sodium azide (SA), mitomycin C (MMC), benzo[*a*]pyrene (B[*a*]P), aflatoxin B_1_ (AFB_1_), 2-amino-anthracene (2-AA) and 2-amino-fluorene (2-AF) were purchased from Sigma Chemical Co. (St. Louis, MO, USA). Doxorubicin (DXR) was purchased from Pharmacia Brasil Ltda, Brazil, and dissolved in distilled water immediately before treatment. Oxoid Nutrient Broth No. 2 (Oxoid, England) and Difco Bacto Agar (Difco, USA) were used as bacterial media. D-glucose, magnesium sulfate, citric acid monohydrate, anhydrous dibasic potassium phosphate, sodium ammonium phosphate, monobasic sodium phosphate, dibasic sodium phosphate and sodium chloride were purchased from Merck (Whitehouse Station, NJ, USA).

### Cell line and culture conditions

Chinese hamster lung fibroblasts (V79) were kindly supplied by Professor Cólus (Universidade Estadual de Londrina (UEL), Paraná, Brazil). Cells were maintained as monolayers in plastic culture flasks (25 cm^2^) in HAM-F10 (Sigma-Aldrich) plus DMEM (Sigma-Aldrich, 1:1) culture medium supplemented with 10%; fetal bovine serum (Nutricell), antibiotics (0.01 mg mL^−1^ streptomycin and 0.005 mg mL^−1^ penicillin; Sigma-Aldrich) and 2.38 mg mL^−1^ Hepes (Sigma-Aldrich), at 37°C in a BOD-type chamber. Under these conditions, the average cell cycle time was 12 h.

### Comet assay

The protocol for the determination of the genotoxicity and antigenotoxicity of HK at various concentrations (0.5 - 128 μM) was performed in triplicate on three different days, to ensure reproducibility. HK was first dissolved in a mixture of methanol (100 μL) and distilled water (900 μL). The final concentration of methanol in the culture was 0.1%;. The choice of concentrations was based on the results of previous experiments with HK [12]. In the experiments, 3 × 10^5^ cells (V79) were seeded into tissue-culture flasks, incubated for two cycles (24 h) in complete HAM-F10/DMEM medium, washed with phosphate buffer saline (PBS), and then subjected to one of the following treatments, in serum-free medium, for 3 h. To assess genotoxicity, the cells were treated with each concentration of HK alone, while for antigenotoxicity, they were treated with the mutagen DXR (0.3 μM) in combination with each HK concentration. Positive (DXR) and negative controls were also included in the test. At the end of the treatment, the cells were washed with ice-cold PBS and trypsinized with 200 μL trypsin. After 3 min, the cells were gently resuspended in complete medium and 20 μL of the cell suspension was immediately used for the test.

The procedures described by Singh et al. [14] were adopted, with minor modifications, as described in detail by Speit and Hartmann [15] and reviewed by Burlinson et al. [16]. Briefly, a microscope slide was covered with a base layer of 1.5%; normal-melting agarose (Invitrogen) and 20 μL of the test cells suspended in 120 μL 0.5%; low-melting agarose (Invitrogen) at 37°C was then spread over the base layer. A coverslip was added and the agarose allowed to solidify at 4°C for 15 min. Next, the coverslip was gently removed and the slides were immersed in freshly prepared lysing solution consisting of 89 mL stock solution (2.5 M NaCl, 100 mM EDTA, 10 mM Tris, pH 10.0, and 1%; sodium lauryl sarcosine), 10 mL DMSO and 1 mL Triton X-100, pH 10.0, at 4°C, for at least 20 h, protected from light. At the end of this period, the slides were transferred to an electrophoresis chamber containing a high pH (>13.0) buffer (300 mM NaOH, 1 mM EDTA) and incubated at 4°C for 20 min to allow the DNA to unwind. A current of 25 V (1.0 V cm^−1^, 300 mA) was applied for 20 min. The slides were then submerged in a neutralization buffer (0.4 M Tris - HCl, pH 7.5) for 15 min, dried at room temperature and fixed in 100%; ethanol for 10 min.

The slides were stained with 100 μL ethidium bromide (20 μg ml^−1^) and covered with a coverslip. All the slides in the experiment were coded before analysis. The stained nucleoids were immediately evaluated at 1000x magnification under a Nikon fluorescence microscope fitted with a 515–560 nm excitation filter and a 590 nm barrier filter.

For each treatment, the extent and distribution of DNA damage indicated by the comet assay were evaluated by examining 100 randomly selected and non-overlapping cells on the slides (i.e. 300 nucleoids per treatment). For each slide, the cells were visually scored and allocated to one of four classes (0, 1, 2 and 3), according to tail size, as follows: class 0, undamaged, no tail; class 1, a short tail whose length was smaller than the diameter of the comet head (nucleus); class 2, tail length between 1 and 2 times the diameter of the head; and class 3, maximally damaged: a long tail measuring more than twice the diameter of the head. The few comets containing no head and those with almost all DNA in the tail, or with a very wide tail, were excluded from the analysis since they may arise from dead cells [17].

The total score for 300 comets was calculated by the formula shown below:

(1) $$Score=\left(1\times n_{1}\right)+\left(2\times n_{2}\right)+\left(3\times n_{3}\right)$$

where *n* = number of cells in each class analyzed. Thus, the total score ranged from 0 to 300. The percentage reduction of genotoxic agent-induced damage by HK was calculated as in Waters et al. [18], with the following formula:

(2) %Reduction=A−B/A−C×100

where *A* is the mean score in the treatment with DXR (positive control), *B* the mean score in the antigenotoxic treatment (HK plus DXR) and *C* the mean score in the negative control.

Cell viability was evaluated for each treatment by Trypan blue staining. Briefly, a solution of 50 μL Trypan blue (0.4%;) freshly prepared in distilled water was mixed with 50 μL of each cell suspension, spread onto a microscope slide and covered with a coverslip. Non-viable cells appeared blue. At least 200 cells were counted per culture.

The results were evaluated by analysis of variance (ANOVA) and the Tukey test at *P* < 0.05, the experimental criterion being the significance of the response to HK treatment in relation to the negative control, in the genotoxicity assay, and in relation to the positive control when the antigenotoxicity of HK was determined as its capacity to reduce the DNA damage induced by DXR.

### Ames test

Mutagenic activity was evaluated by the *Salmonella*/ microsome assay, using the *Salmonella typhimurium* tester strains TA98, TA100, TA97a and TA102, kindly provided by Dr. B.N. Ames (Berkeley, CA, USA), with (+ S9) and without (− S9) metabolization, by the pre-incubation method [19]. The strains were grown from frozen cultures overnight for 12–14 h in Oxoid Nutrient Broth No. 2. The metabolic activation mixture (S9 fraction), prepared from livers of Sprague–Dawley rats treated with the polychlorinated biphenyl mixture Aroclor 1254 (500 mg/ kg), was purchased from Molecular Toxicology Inc. (Boone, NC, USA) and freshly prepared before each test. The metabolic activation system consisted of 4%; S9 fraction, 1%; 0.4 M MgCl_2_, 1%; 1.65 M KCl, 0.5%; 1 M D-glucose-6-phosphate disodium and 4%; 0.1 M NADP, 50%; 0.2 M phosphate buffer and 39.5%; sterile distilled water [19].

For the determination of the mutagenic activity, five different concentrations of HK (9.75 – 78.0 μg∕ plate), diluted in DMSO, were assayed. The concentrations of HK were selected on the basis of a preliminary toxicity test. In all subsequent assays, the upper limit of the dose range tested was either the highest non-toxic dose or the lowest toxic dose determined in this preliminary assay. Toxicity was detected either as a reduction in the number of histidine revertants (His+), or as a thinning of the auxotrophic background (*i*.*e*., background lawn). The various concentrations of HK to be tested were added to 0.5 mL of 0.2 M phosphate buffer, or to 0.5 mL of 4%; S9 mixture, with 0.1 mL of bacterial culture and then incubated at 37°C for 20–30 min. Next, 2 mL of top agar was added and the mixture poured on to a plate containing minimal agar.

The plates were incubated at 37°C for 48 h and the His+ revertant colonies were counted manually. All experiments were analyzed in triplicate. The results were analyzed with the statistical software package Salanal 1.0 (U.S. Environmental Protection Agency, Monitoring Systems Laboratory, Las Vegas, NV,from Research Triangle Institute, RTP,NC, USA), adopting the Bernstein et al. [20] model. The data (revertants/ plate) were assessed by analysis of variance (ANOVA), followed by linear regression. The mutagenic index (MI) was also calculated for each concentration tested, this being the average number of revertants per plate with the test compound divided by the average number of revertants per plate with the negative (solvent) control. A test solution was considered mutagenic when a dose–response relationship was detected and a two-fold increase in the number of mutants (MI ≥ 2) was observed for at least one concentration [21]. The standard mutagens used as positive controls in experiments without S9 mix were NOPD (10 μg/ plate) for TA98 and TA97a, SA (1.25 μg/ plate) for TA100 and MMC (0.5 μg/ plate) for TA102. In experiments with S9 activation, 2-AA (1.25 μg /plate) was used with TA98, TA97a and TA100 and 2-AF (10 μg/ plate) with TA102. DMSO (50 μL/ plate) served as the negative (solvent) control.

The antimutagenicity assay was conducted by means of the same procedure as the mutagenicity assay, except that HK was associated with known mutagens in tests with and without metabolic activation. In these tests, the direct-acting mutagens were 10.0 μg/ plate of NOPD (for *S*. *typhimurium* TA98 and TA97a), 1.25 μg/ plate of SA (for *S*. *typhimurium* TA100) and 0.5 μg/ plate of MMC (for *S*. *typhimurium* TA102), in the assay without metabolic activation, and the indirect-acting mutagens were 1.0 μg/ plate of B[*a*]P (for *S*. *typhimurium* TA98), 0.5 μg/ plate of AFB_1_ (for *S*. *typhimurium* TA100), 1.25 μg/ plate of 2-AA (for *S*. *typhimurium* TA97a) and 10 μg/ plate of 2-AF (for *S*. *typhimurium* TA102), in the assay with metabolic activation. All the plates were incubated at 37°C for 48 hours, and the number of revertant colonies per plate was counted manually. The entire assay was performed in triplicate.

The antimutagenicity results were expressed as percent inhibition (the ability of the compounds to inhibit the action of the known mutagen). This was calculated as follows:

(3) $$Inhibition\left(\%\right)=100-\left[\left(T/M\right)\times 100\right]$$

where *T* is the number of revertant colonies in the plate containing mutagen and compounds and *M* is the number of revertant colonies in the plate containing only the mutagen [22].

No antimutagenic effect was recorded when the inhibition was lower than 25%;, a moderate effect for a value between 25%; and 40%;, and strong antimutagenicity for values greater than 40%; [23,24].

Cell viability was also determined for each antimutagenesis experiment, to assess the potential bactericidal effect of the mutagens. A substance was considered bactericidal when the bacterial survival was less than 60%; of that observed in the negative control [24,25].

## Results

### Comet assay

The results for V79 cells treated with HK by comet assay are shown in Table 1. No significant difference was observed between cultures treated with HK and the negative control group (*P*>0.05), demonstrating the absence of genotoxicity. On the other hand, a significant increase in the rate of DNA damage was observed in cultures treated with DXR, relative to the negative control, as expected.

**Table 1:** DNA migration in the comet assay from V79 cells treated with various doses of HK and/or DXR and their respective controls

**Treatments(μM)**	Class^*^				Score^*^	**Reduction %;**
	**0**	**1**	**2**	**3**		
Control	84.3 ± 9.0	10.0 ± 4.0	2.3 ± 2.0	1.6 ± 2.0	20.3 ± 13.0	-
MeOH	87.0 ± 7.0	11.0 ± 5.5	2.3± 1.5	0	18.0 ± 5.1	-
0.5	81.6 ± 4.9	12.3± 3.5	4.3 ± 3.7	5.0 ± 2.8	26.0 ± 13.2	-
1.0	87.0 ± 5.1	11.0 ± 5.2	1.3 ± 0.5	0	13.0 ± 5.0	-
2.0	83.0 ± 3.7	14.0 ± 2.5	2.0 ± 2.0	0	18.0 ± 5.0	-
32	89.0 ± 2.0	10.0 ± 2.0	0.3 ± 0.5	0.6 ± 1.1	9.6 ± 2.3	-
64	88.0 ± 3.6	11.0 ± 4.0	0.6 ± 1.0	1.0 ± 1.0	13.0 ± 3.4	-
128	89.0 ± 1.5	10.0 ± 1.5	1.0 ± 0.0	0	12.0 ± 1.5	-
DXR	41.0 ± 12.0	36.0 ± 12.7	18.0 ± 2.0	4.0 ± 2.5	86.0 ± 11.5^a^	-
DXR + MeOH	46.3 ± 6.8	30.0 ± 6.0	16.3 ± 5.6	6.6 ± 3.0	83.3 ± 14.5^a^	-
0.5 + DXR	73.0 ± 7.0	20.0 ± 7.5	4.6 ± 1.5	2.0 ± 2.0	36.0 ± 6.1^a,b^	76.0
1.0 + DXR	68.0 ± 2.0	20.6 ± 2.5	8.0 ± 2.0	3.3 ± 0.5	46.0 ± 3.0^a,b^	60.8
2.0 + DXR	65.0 ± 6.8	27.0 ± 6.6	4.3 ± 2.3	2.6 ± 1.6	44.3 ± 9.2^a,b^	63.4
32 + DXR	35.0 ± 5.0	33.0 ± 3.0	30.0 ± 4.1^b^	1.0 ± 1.0	97.0 ± 10.0^a^	-
64 + DXR	32.0 ± 6.1	39.0 ± 3.7	26.0 ± 2.0	2.0 ± 2.0	97.0 ± 10.0^a^	-
128 + DXR	28.0 ± 6.6	40.0 ± 1.0	29.0 ± 5.2^b^	3.0 ± 2.5	106.0 ± 16.0^a^	-

HK = (−)-hinokinin; MeOH= methanol (0.1%;); DXR= doxorubicin (0.3 μM).

The different doses (0.5, 1.0, 2.0, 32, 64 and 128 μM) correspond to HK.

^*^Values are the means ± standard deviation.

^a^ Significantly different from control (*P*<0.05).

^b^ Significantly different from the DXR group (*P*<0.05).

In the treatments with HK associated with DXR, the lower concentrations of HK (0.5; 1.0 and 2.0 μM) significantly reduced the extent of DNA damage induced by DXR. This significant reduction in the frequency of DNA damage ranged from 60.8 to 76.0%;. The gradual increase in the concentration of HK did not lead to a proportional increase in the reduction of DXR-induced genotoxicity, thus demonstrating the absence of a dose–response relationship (Table 1).

However, at the higher concentrations of HK (32; 64 and 128 μM) associated with DXR, the extent of DNA damage did not differ significantly from the frequencies observed in the DXR treatment. The data also showed that the extent of class 2 damage was higher in treatments with HK and DXR than in the group treated with DXR, which was statistically significant at concentrations of 32 and 128 μM (Table 1).

Comet class 0 was the most frequent among cultures treated with various doses of HK, negative and solvent controls, and lower concentrations of HK plus DXR, whereas comet classes 1 and 2 were the most frequent among cultures treated with only with DXR or MeOH plus DXR (Table 1). Cell viability was higher than 95%; in all treatments.

There was no significant difference in the extent of DNA damage between cultures treated with the solvent plus DXR and the positive control.

### Ames test

Table 2 shows the mean number of revertants/plate (M), the standard deviation (SD) and the mutagenic index (MI) after the treatments with HK, observed in *S*. *typhimurium* strains TA98, TA100, TA102 and TA97a, in the presence (+S9) and absence (−S9) of metabolic activation. The mutagenicity assays show that HK did not induce any increase in the number of revertant colonies relative to the negative control, indicating the absence of any mutagenic activity.

**Table 2:** **Revertants/ plate, standard deviation and mutagenicity index (in brackets) for the strains TA98, TA100, TA102 and TA97a of *S*. *typhimurium *after treatment with various doses of HK, with (+S9) and without (−S9) metabolic activation**

	**Treatments**	**Number of revertants (M±SD)/ plate and MI**							
	**μg**/**plate**	**TA 98**		**TA 100**		**TA 102**		**TA 97a**	
		**- S9**	**+ S9**	**- S9**	**+ S9**	**- S9**	**+ S9**	**- S9**	**+ S9**
**HK**	**0**.**0**^**a**^	22 ± 2	32 ± 1	132 ± 6	123 ± 1	243 ± 4	391 ± 8	164 ± 4	163 ± 2
	**9**.**7**	18 ± 4 (0.8)	32 ± 1 (1.0)	134 ± 4 (1.0)	130 ± 6 (1.1)	238 ± 8 (1.0)	376 ± 7 (1.0)	165 ± 5 (1.0)	172 ± 10 (1.0)
	**19**.**5**	23 ± 3 (1.0)	34 ± 3 (1.1)	129 ± 6 (1.0)	128 ± 6 (1.0)	231 ± 8 (0.9)	363 ± 3 (0.9)	165 ± 9 (0.9)	187 ± 3 (1.1)
	**39**.**0**	25 ± 3 (1.1)	33 ± 4 (1.0)	134 ± 5 (1.0)	115 ± 8 (0.9)	213 ± 4 (0.9)	354 ± 4 (0.9)	146 ± 5 (0.9)	166 ± 5 (1.0)
	**58**.**5**	25 ± 2 (1.1)	28 ± 1 (0.8)	112 ± 3 (0.8)	134 ± 2 (1.1)	201 ± 6 (0.8)	398 ± 9 (1.0)	175 ± 4 (1.1)	172 ± 2 (1.0)
	**78**.**0**	24 ± 5 (1.1)	31 ± 2 (1.0)	140 ± 9 (1.1)	120 ± 8 (1.0)	186 ± 2 (0.8)	402 ± 3 (1.0)	163 ± 8 (1.0)	146 ± 4 (0.9)
	**Ctrol** +	1347 ± 88^b^	1567 ± 115^e^	1582 ± 98^c^	1456 ± 78^e^	1656 ± 60^d^	1932 ± 97^f^	1766 ± 49^b^	1789 ± 89^e^

HK = (−)-Hinokinin; M ± SD = mean and standard deviation; MI = mutagenicity index; ^a^Negative control: dimethylsulfoxide (DMSO - 50 μL/ plate); Ctrol + = Positive control - ^b^4 -nitro-*o*-phenylenediamine (NOPD – 10.0 μg/ plate – TA98, TA97a); ^c^sodium azide (1.25 μg/ plate – TA100); ^d^mitomycin (0.5 μg/ plate – TA102), in the absence of S9 and ^e^2-anthramine (1.25 μg/ plate – TA 97a, TA98, TA100); ^f^2-aminofluorene (10.0 μg/ plate – TA102), in the presence of S9.

On the other hand, the results obtained in the tests for antimutagenic potential of HK, presented in Table 3, show a strong inhibitory effect against direct and indirect-acting mutagens, for strains TA98, TA100, TA102 and TA97a. The results are expressed as mean number of revertants/ plate (M), the standard deviation (SD) and the percent inhibition of mutagenic activity of a sample containing a mixture of mutagen and HK, relative to the mutagenicity of the mutagen alone.

**Table 3:** **Antimutagenic activity expressed as the mean and standard deviation of number of revertants and percent inhibition by HK of direct (−S9) and indirect (+S9) mutagens, tested on strains TA98, TA100, TA102 and TA 97a of *S*. *typhimurium***

**Treatments**	**Number of revertants(M ± SD)/ plate and %; of inhibition**							
**HK**	**TA 98**				**TA 100**			
**μg**/**plate**	− **S9**	%; **inhibition**	+ **S9**	%; **inhibition**	− **S9**	%; **inhibition**	+ **S9**	%; **inhibition**
**Ctrol** +	**NOPD**		**B**[***a***]**P**		**SA**		**AFB**_**1**_	
	638 + 30		1244 + 38		1219 + 46		1607 + 79	
**9**.**7**	525 + 7	18*	874 + 21	31**	1139 + 20	7*	1183 + 30	30**
**19**.**5**	507 + 15	21*	869 + 4	32**	1112 + 34	9*	976 + 18	45***
**39**.**0**	477 + 5	26**	813 + 18	36**	1169 + 32	5*	943 + 11	47***
**58**.**5**	521 + 9	19*	618 + 6	52***	1145 + 16	7*	612 + 33	71***
**78**.**0**	480 + 16	25**	539 + 32	59***	1181 + 41	3*	354 + 22	89***
	**TA 102**				**TA 97a**			
**μg**/**plate**	- **S9**	%; **inhibition**	+ **S9**	%; **inhibition**	- **S9**	%; **inhibition**	+ **S9**	%; **inhibition**
**Ctrol** +	**MMC**		**2**-**AF**		**NOPD**		**2**-**AA**	
	1184 + 42		1279 + 12		884 + 34		1083 + 67	
**9**.**7**	1236 + 17	-	852 + 21	42***	934 + 33	-	682 + 52	43***
**19**.**5**	1201 + 23	-	855 + 22	42***	948 + 24	-	659 + 41	45***
**39**.**0**	1317 + 14	-	820 + 19	46***	979 + 84	-	628 + 16	49***
**58**.**5**	1017 + 56	18*	754 + 9	52***	1006 + 54	-	456 + 13	67***
**78**.**0**	979 + 51	22*	757 + 29	52***	1026 + 75	-	523 + 86	60***

HK = (−)-Hinokinin; M ± SD = mean and standard deviation; Ctrol + = positive Control; NOPD = 4 -nitro-*o*-phenylenediamine (10.0 μg/ plate – TA98 and TA97a); SA = sodium azide (1.25 μg/ plate – TA100); MMC = mitomycin (0.5 μg/ plate – TA102), in the absence of S9 and B[*a*]P= benzo[*a*]pyrene (1.0 μg/ plate – TA 98); AFB_1_ = aflatoxin B_1_ (0.5 μg/ plate – TA 100); 2-AA = 2-anthramine (1.25 μg/ plate – TA 97a); 2-AF = 2-aminofluorene (10.0 μg/ plate – TA102), in the presence of S9.

* no antimutagenic effect (< 25%; inhibition).

** moderate effect (25%; - 40%; inhibition).

*** strong antimutagenic effect (> 40%; inhibition).

When strain TA98 was used in association with NOPD, a moderate antimutagenic effect was observed for HK (26%; inhibition). In experiments with metabolic activation, for strain TA98, the mutagenicity of B[*a*]P was significantly reduced by 59%;.

HK did not reduce mutagenesis induced by SA, MMC or NOPD, in the absence of metabolic activation, when strains TA100, TA102 and TA97a were used, respectively. However, HK did inhibit mutation induced by the alkylating agent AFB_1_ in TA100, produced significant decreases in the mutagenicity of 2-AF in TA102 (52%;) and a strong antimutagenic effect against mutations induced by 2-AA in TA97a (67%; inhibition). The highest observed percent inhibition of mutagenicity (89%;) achieved with HK was in strain TA100, in the presence of AFB_1_. Furthermore, HK potentiated NOPD -induced clastogenicity in the strain 97a: the number of revertents observed for the combined treatment was higher than that observed for the positive control alone.

## Discussion

The balance between the therapeutic and toxicological effects of a compound is a very important measure of the usefulness of a pharmacological drug. Therefore, the determination of the potential mutagenic effect of any drug under development is mandatory [26].

In previous studies, Medola et al. [1] showed that HK not only had no genotoxic effect, but also was effective in reducing the chromosome damage induced by DXR, by the rat peripheral blood micronucleus test. Recently, Resende et al. [12] assessed the possible genotoxic activity of HK and its influence on the activities of two known mutagenic agents (DXR and methyl methanesulfonate - MMS), in the micronucleus test with Chinese hamster lung fibroblast V79 cells. HK alone had no genotoxic effect under the conditions tested, but it reduced the chromosome damage caused by MMS. The reduction in DXR-induced clastogenicity was observed at lower concentrations. At higher concentrations, HK acted as a potentiator of DXR-induced clastogenicity, with the observation of a significantly higher frequency of micronuclei in the combined treatment when compared to the positive control.

To complement the above results, the genotoxic∕ mutagenic activities of HK, and its influence on the activities of known mutagenic agents, were assessed by comet and Ames test in this study. According to Witte et al. [27], experience with genetic toxicology testing over the past few decades has demonstrated that no single test method is capable of detecting all types of genotoxic effects. Therefore, the potential for a chemical to cause genotoxicity is typically determined by using a battery of *in vitro* and *in vivo* tests.

Through the comet assay, the first and extremely important observation was the absence of DNA strand breaks; moreover, there were no gene mutations by the Ames test in the presence and absence of metabolic activation. The performance of assays for to assess mutagenicity, as well as other risks, is essential, given the potential consumption of HK by the population. The absence of genotoxic∕ mutagenic effects by HK on V79 cells in the comet test and against *S*. *typhimurium* bacterial strains in the Ames test is a positive step towards ensuring its safe use in medicine. Considering the possible use of HK as an antichagasic drug, a lack of mutagenic effects in animal cells and bacteria is highly relevant.

On the other hand, the influence of HK on DXR-induced DNA damage depends on the experimental conditions used and draws attention to the synergistic effect that HK may have when combined with other drugs. In the comet test, the lower concentrations of HK (0.5; 1.0 and 2.0 μM) significantly reduced the extent of DNA damage induced by DXR. However, the higher concentrations of HK (32; 64 and 128 μM), when combined with DXR, showed a higher rate of class 2 damage than in the cells treated with DXR, which was statistically significant at concentrations of 32 and 128 μM. However, the extent of DNA damage did not differ significantly from the frequencies observed in the DXR treatment. These results are consistent with Resende et al. [12], who assessed the influence of HK, at the same concentrations, on DXR-induced genotoxicity.

The chemical structure of DXR favors the generation of free radicals and the compound can bind to iron and form complexes with DNA, inducing DNA damage. Some studies have demonstrated that oxidative damage is probably related to this formation of free radicals accompanied by a reduction in antioxidant capacity [28]. Thus, at low concentrations, HK might possibly interfere in the intercalation of DXR with DNA or scavenge the generated free radicals. However, at higher doses, HK may increase the oxidative stress generated by DXR, since qualitative HPLC analysis showed that no new compound is formed after the incubation of a mixture of DXR and HK. HK may act as a “janus” compound, i.e., exerting an antioxidant effect at lower concentrations and a pro-oxidant effect at higher concentrations [12]. The synergistic effect also was observed when HK was combined with NOPD in the strain TA97a in the absence of metabolic activation in the Ames test, reinforcing the hypothesis that the HK may act as a “janus” compound.

In the antimutagenicity evaluated by Ames test, HK exhibited a protective effect in more than one test strain and acted against various mutational mechanisms. Among the antimutagenic activity against directly acting mutagens, a moderate effect was found only against frameshift mutations induced by NOPD in the TA98 strain, with the highest %; of inhibition at concentration of 39.0 μg/ plate (26%;).

HK did not affect the SA-induced mutagenicity in strain TA100, MMC in strain TA102 or NOPD in strain 97a.

The protection of the bacterial genome against directly acting mutagens may be due to the rapid elimination of mutagens from the bacteria, before their interaction with the DNA [29]. HK may facilitate or stimulate the bacterial transmembrane export system to eliminate the mutagens; it may also interfere with the uptake of mutagens into bacteria [29,30].

The activity displayed by HK was profoundly increased by incorporating the microsomal fraction (S9), which is a mammalian metabolic activation system, into the culture medium. The results of this experiment show that HK inhibited B*a*P, AFB_1_, 2-AF and 2-AA mediated mutagenesis. The microsomal fraction of rat liver, containing mixed-function oxidase (MFO) and the cytochrome-based P450 metabolic oxidation system, can activate B*a*P to an active mutagen, benzo*a*pyrene-7,8-diol-9,10-epoxide [31]. The mutagenicity of B*a*P was significantly reduced in a dose-dependent manner by 31 to 59%; by HK.

This diol epoxide exerts its carcinogenic activity by alkylating nucleosides on DNA molecules at their bay region. The reaction occurs primarily with the purine bases, deoxyguanosine and deoxyadenosine, in DNA [32]. As a result, bulky stable and depurinating DNA adducts are formed [33,34]. Insufficient removal of these DNA adducts prior to replication creates hot spots in the gene and can result in deactivation of tumor suppressor genes or activation of oncogenes leading to tumor initiation [35,36].

There are at least two possible mechanisms by which HK could decrease B*a*P-DNA adduct formation: by interacting with reactive intermediates or by interfering with the action of microsomal enzymes [36]. However, more studies are needed to confirm these ideas.

HK also reduced the frequency of mutations induced by the fungal toxin, AFB_1_, in TA100 with metabolic activation, resulting in the highest percent inhibition of mutagenicity (89%;). The *S*. *typhimurium* tester strain TA100 reveals base-pair-substitution point mutations [37].

Aflatoxins, a group of potent mycotoxins with mutagenic, carcinogenic, teratogenic, hepatotoxic and immunosuppressive properties, are of particular importance because of their adverse effects on animal and human health. Aflatoxins are produced as secondary metabolites by fungi of various species of *Aspergillus* (*A*. *flavus*, *A*. *parasiticus* and *A*. *nomius*) that grow on a variety of food and feed commodities. AFB_1,_ which is the most toxic aflatoxin, is metabolized mainly in the liver to AFB_1_-8,9-exo-epoxide and 8,9-endo-epoxide. The exo-epoxide form of AFB_1_ binds to DNA to form the predominant 8,9-dihydro- 8-(N7-guanyl)-9-hydroxy AFB_1_ adduct, leading to a more stable imidazole ring-opened AFB_1_–formamidopyrimidine adduct. The pseudo-half-life for loss of 8,9-dihydro-8- (N7-guanyl)-9-hydroxy AFB_1_ is short, but AFB_1_–formamidopyrimidine adducts are stable, accumulate for several days and remain detectable for several weeks [38]. This aflatoxin is of particular interest because it is a frequent contaminant of many food products and one of the most potent naturally occurring mutagens and carcinogens known [39].

HK also induced a strong antimutagenic effect, significantly diminishing the mutagenicity of 2-AF in TA102 with metabolic activation, in a dose-dependent manner, by 42 to 52%;. 2-AF is converted in rat liver, via *N*-hydroxy metabolites, to the reactive carcinogenic ester 2-acetylaminofluorene-*N*-sulfate, which can attack guanine residues in nucleic acids [31]. The inhibition of 2-AF induced mutagenicity may be mediated through the inhibition of the MFO (in the S9 fraction) or inactivation of the activated reactive ester of 2-AF. The *S*. *typhimurium* tester strain TA102 is normally used to detect mutagens that cause oxidative damage and base-pair-substitution mutations [37]. In this case, antimutagenic activity can be partially ascribed to antioxidant activity. This speculation is further supported by the significant antimutagenic effect that the lower concentrations of HK demonstrated against DXR in the comet test, as well as that against mutagens needing metabolic activation, where free radical generation is anticipated.

In this study, the antimutagenic property of HK related to its ability to modulate the xenobiotic-metabolizing enzymes in the liver, either by preventing the metabolic activation or by altering the enzymatic activity in the detoxification pathway to induce the disposal of the known mutagen [22], was again demonstrated by the results obtained with the mutagen 2-AA in strain TA97a with metabolic activation, where 67%; inhibition was observed.

In general, inhibitors of mutagenesis can act in one of several ways: by inhibiting the interaction between genes and biochemically reactive mutagens; inhibiting metabolic activation of indirectly-acting mutagens by inactivation of metabolizing enzymes, or interacting with the pro-mutagens to make them unavailable for the enzymatic process [40].

## Conclusions

In view of the above results and hypotheses, we can state that the inhibition of mutagenesis is often complex and involves multiple mechanisms. These results emphasize that antimutagenic mechanisms of HK cannot be generalized and that it is worth investigating each of them independently. The importance of this study is that HK no had to genotoxic / mutagenic effect in the comet and Ames assays and the DNA protective activity of HK is not general, therefore, this study demonstrates that besides the therapeutic potential for trypanosome diseases free of genotoxic / mutagenic effect, HK can provide a benefit antimutagenic effect, depending of the type of DNA damage-inducing agent used.

## Abbreviations

HK: (−)-hinokinin; NOPD: 4- nitro-*o*-phenylenediamine; SA: Sodium azide; MMC: Mitomycin C; B[*a*]P: Benzo[*a*]pyrene; AFB_1_: Aflatoxin B_1_; 2-AA: 2-aminoanthracene; 2-AF: 2-aminofluorene; DXR: Doxorubicin; DMSO: Dimethylsulfoxide; PBS: Phosphate buffer saline; V79: Chinese hamster lung fibroblasts; +S9: With metabolization; –S9: Without metabolization; MI: Mutagenic index.

## Competing interests

The authors declare that they have no competing interests.

## Authors’ contributions

FAR designed and performed the experiments, interpreted the results and drafted the manuscript. LCB and DCT designed and performed the comet assay. MSC participated in the experiments of the Ames test. KCSR and MLAS isolated the (−)-cubebin and prepared the (−)-hinokinin by partial synthesis. EAV critically read the manuscript and participated in revision of the manuscript. All authors have read and approved the final manuscript.

## Pre-publication history

The pre-publication history for this paper can be accessed here:

http://www.biomedcentral.com/1472-6882/12/203/prepub
